# Reactive Hyperemia Index in Patients on Maintenance Hemodialysis: Cross-sectional Data from a Cohort Study

**DOI:** 10.1038/srep45757

**Published:** 2017-03-30

**Authors:** Wenjin Liu, Meijuan Meng, Jianping Chen, Liang Wang, Zhuxing Sun, Xiurong Li, Jianmei Zhou, Chaoqing Gao, Jiajun Zhou, Hong Chu, Wei Fan, Youwei Bai, Junwei Yang

**Affiliations:** 1 Center for Kidney Disease, Second Affiliated Hospital of Nanjing Medical University, Nanjing, China; 2 Department of Statistics Analysis, Affiliated Drum Tower Hospital, Nanjing University Medical School, Nanjing, China; 3 Department of Nephrology, Wuxi People’s Hospital, Nanjing Medical University, Wuxi, China; 4 Department of Blood Purification, The Third Affiliated Hospital of Soochow University, Changzhou, China; 5 Department of Hemodialysis, Yijishan Hospital of Wannan Medical College, Wuhu, China; 6 Department of Nephrology, Affiliated Yixing People’s Hospital, Jiangsu University, Yixing, China; 7 Department of Nephrology, Luan People’s Hospital, Luan, China

## Abstract

Previous studies suggested that the reactive hyperemia index (RHI) is a promising cardiovascular risk predictor. We aimed to evaluate clinical determinants of RHI and its association with circulating endothelial injury and cardiac markers in hemodialysis patients. Among 368 patients recruited, RHI was evaluated by peripheral arterial tonometry (PAT) on a midweek nondialysis day. Clinical determinants of RHI were explored by multiple stepwise regression analysis and associations between RHI and circulating markers were evaluated by general linear models. The major cause of a failed PAT test was poor signal (82.1%). Intraclass correlation coefficient for reproducibility evaluation was 0.74. Multiple regression analysis showed traditional clinical factors only explained 7% of the variance of natural logarithm RHI (LnRHI) in the patients. In association analyses, LnRHI showed significant positive associations with Von Willebrand factor (vWF) (*p* = 0.04) and tissue factor (*p* = 0.047). It also associated positively with troponins (*p* ≤ 0.02 for both). In conclusion, performance of the PAT test was acceptable in dialysis patients and traditional clinical variables had very limited influence on RHI in these subjects. Among a panel of conventional endothelial injury markers, RHI showed very modest associations with only vWF and tissue factor. RHI associated positively with troponins in the patients.

Patients with kidney disease suffer from an increased risk of cardiovascular events as renal function declines^1^. For those on maintenance hemodialysis, cardiovascular disease remains the leading cause of death^2^. This high prevalence and severity of cardiovascular injury in these patients is believed to be induced by both traditional and non-traditional risk factors, of which endothelial dysfunction is suggested to play an important role^3^. Endothelial dysfunction is a dominant feature of atherogenesis and a key aspect in the development of cardiovascular diseases. It is characterized primarily by reduced bioactivity of nitric oxide (NO), which is essential to the regulation of vascular tone and homeostasis^4^.

In clinical practice and research studies, the most common technique to evaluate endothelial function is flow-mediated vasodilatation (FMD) of the brachial artery. Previous studies have demonstrated that endothelial function evaluated by this method correlate closely with results measured directly in the coronary artery^5^,^6^. However, its application in practice requires a highly experienced operator, and comparability in different settings is challenging^7^. Recently, a new device (EndoPAT 2000) using peripheral arterial tonometry (PAT) provides an alternative option for non-invasive measurement of endothelial function. Placed on a fingertip, the device measures pulse arterial volume changes induced by upper arm cuff occlusion and generates the reactive hyperemia index (RHI) automatically. Unlike the FMD measurement, RHI is operator-independent and easy to perform. Several studies have validated its value in assessing coronary microvascular endothelial function and in predicting cardiovascular outcome in patients at risk, including those with chronic kidney disease^8^,^9^,^10^.

A previous study has adopted the index as marker of endothelial function in a group of peritoneal dialysis patients^11^. However, there is a paucity of data regarding RHI in patients on hemodialysis, in spite of the fact that these patients bear a significant burden of cardiovascular disease. Exploring non-traditional and novel risk indicators is of great importance for these individuals. In addition, the presence of an arteriovenous fistula in these patients may influence the test results as well as its clinical relevance. Here, we explored the performance of the test in dialysis patients to evaluate its clinical utility with a focus on: 1). clinical determinants of this index and 2). its associations with traditional endothelial injury markers and cardiac risk markers.

## Subjects and Methods

### Study Population

We performed this cross-sectional analysis using baseline data from an ongoing prospective cohort study, which aims to study vascular dysfunction in dialysis patients. The study included patients on maintenance hemodialysis (four hours per treatment and thrice weekly over 3 months) and aged 18–80 years. All patients reached their dry weight based on clinical judgement. Exclusion criteria included: 1. Malignant hypertension with systolic blood pressure (SBP) ≥180 mmHg or diastolic blood pressure (DBP) ≥110 mmHg; 2. Acute infection, acute heart failure or the acute phase of stroke; 3. Decompensated liver cirrhosis or malignant tumor; 4. Amyloidosis or dilated cardiomyopathy; 5. Internal jugular venous catheterization, previous fistula creation on the non-access arm or any other conditions that will affect the accuracy of peripheral arterial tonometry. Coordinating centers of the study included dialysis departments of six tertiary hospitals: The Second Affiliated Hospital of Nanjing Medical University (Nanjing, China), The Third Affiliated Hospital of Soochow University (Changzhou, China), Luan People’s Hospital (Luan, China), Affiliated Yixing People’s Hospital of Jiangsu University (Yixing, China), Affiliated Wuxi People’s Hospital of Nanjing Medical University (Wuxi, China), Yijishan Hospital of Wannan Medical College (Wuhu, China). Sixty age and sex-matched healthy controls (without any known history of chronic kidney disease or cardiovascular disease) were also recruited to assess the difference of RHI between patients and healthy subjects. The study was approved by the Institutional Ethical Committee of The Second Affiliated Hospital of Nanjing Medical University. The study was conducted in accordance with approved guidelines and all participants provided written informed consents.

### Peripheral Arterial Tonometry Measurement

PAT measurement was performed on a midweek nondialysis day. Patients were instructed to abstain from meals, caffeine, and nitrates within 2 hours (long-acting nitrates for 12 hours) before the measurement. Test room temperature was set at 21–25 °C. Peripheral blood pressure was measured at least 20 minutes before the test was started. The EndoPAT 2000 (Itamar Medical Inc., Israel) device was used for the measurement. Specially designed finger probes were placed on the index finger of each hand. Baseline measurement lasted for 6 minutes and followed by occlusion of pulsatile arterial flow for 5 minutes. Occlusion was done through inflation of a blood pressure cuff on the non-access arm, started at a pressure of 250 mmHg and increased until a complete occlusion was achieved as judged by the PAT signal or to a maximum of 300 mmHg. After occlusion, PAT signal was recorded for another 5 minutes. Results were analyzed automatically using an algorithm by the computer^12^. For reproducibility evaluation, a repeated test was performed on the same time of the following week.

### Blood Pressure Measurements

Blood pressure (BP) level was determined by ambulatory blood pressure (ABP) monitoring, which started on the midweek nondialysis day and ended before next dialysis treatment. The SpaceLabs 90217 (SpaceLabs Medical Inc, Redmond, WA) monitor was used and programmed to measure BP every 20 minutes during the day-time (6:00 am.–10:00 pm.) and every 30 minutes during the night-time (10:00 pm.–6:00 am.). Patients were instructed to follow their daily activity and keep their arm immobile during measurements. Recordings were downloaded using manufacturer’s software (SpaceLabs Report Manager System) and were further extracted and analyzed by SPSS 19.0 (SPSS Inc, Chicago, IL). For patients who had <6 ABP readings, their predialysis blood pressures from dialysis records were collected and averaged over 2-weeks (6 times) before the ABP measurements and were used instead of ABP results.

### Laboratory Tests and Biomarker Measurements

Blood samples were drawn through vascular access before dialysis treatment. Routine laboratory tests were performed in each coordinating center. Plasma samples were stored at −80 °C until needed for testing.

Plasma biomarkers, including brain natriuretic peptide (BNP), N-terminal pro-brain natriuretic peptide (NT proBNP), Troponin I, Troponin T and C-reactive protein (CRP), plasminogen activator inhibitor-1 (PAI-1), Von Willebrand factor (vWF), sE-Selectin, tissue factor, thrombomodulin were measured using the Milliplex Map assays (Merck Millipore, Shanghai, China). Concentrations were calculated using the 5-parameter logistic curve fit. Results outside the range of the standards or the fit were further analyzed via cubic spline.

### Statistical Analysis

Data were expressed as mean ± standard deviations or median (interquartile range) for numerical variables and counts (%) for categorical variables. Numerical variables with skewed distribution were logarithm transformed. RHI was expressed in the form of its natural logarithm: LnRHI. Comparisons between two groups were done using the Student’s t test or chi-square test as appropriate. Pearson’s correlation coefficient was used for univariate association analysis and variables with *p* < 0.10 were selected and entered into the following multiple stepwise regression model. Associations between RHI and plasma markers were determined by general linear models. Three models for the association analysis were constructed with different adjustments. The Model-1 was a basic model in which only age and sex were adjusted. In Model-2, major cardiovascular risk factors were included: age, sex, body mass index, smoking status, diabetes mellitus, history of cardiovascular disease, use of antihypertensives, use of statins, total and HDL cholesterol, triglycerides and mean arterial pressure. In Model-3, dialysis-specific risk factors (dialysis vintage and interdialytic weight gain) and log-transformed CRP were added as covariates. All statistical analyses were performed using SPSS 19.0 (IBM SPSS, Chicago, IL). A *p* value < 0.05 was considered statistically significant.

## Results

### General Test Performance and Reproducibility

**Figure 1:**
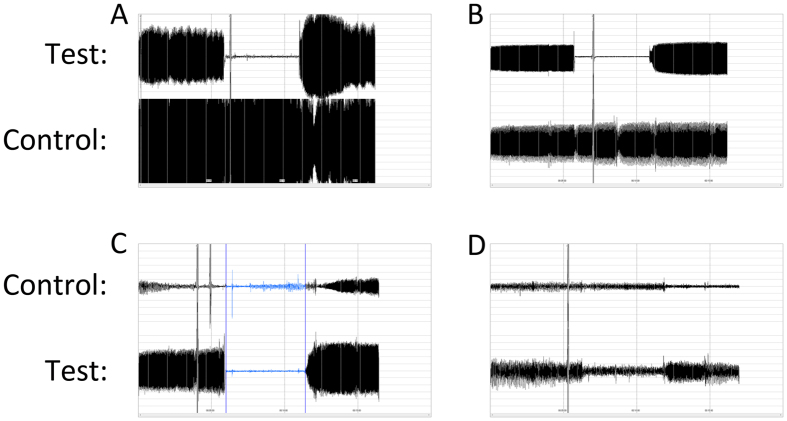
Representatives of Successful and Failed PAT Tests. (**A**,**B**) successful PAT tests for patients with preserved (**A**) and blunted (**B**) response to hyperemia. (**C,D**) failed PAT tests due to poor signal (**C**) and insufficient occlusion (**D**). Note that in D PAT signal of the control arm was also poor.

A total of 368 patients were recruited between July 2015 and July 2016. Eighteen of them did not undergo PAT test due to device availability. Among those who underwent the test, PAT results were available for 311 patients. Causes of a failed test included: poor PAT signal (n = 32, 82.1%), signal with too much noise (n = 1, 2.6%), incomplete occlusion (n = 3, 7.7%), computer breakdown (n = 1, 2.6%), intolerance of occlusion (n = 1, 2.6%) and hand abnormality (n = 1, 2.6%). Representative data from successful and unsuccessful tests are presented in Fig. 1.

Twenty patients were invited to assess reproducibility of testing. Two failed the first test and another two failed the second test. Sixteen had successful first and second tests and the intraclass correlation coefficient was 0.74.

### Characteristics of Patients according to LnRHI Category

General clinical information of the 311 patients who had successful PAT test is presented in Table 1. Mean age of the study population was 52.6 years and 175 (56.3%) were male. Patients with a higher LnRHI had lower body mass index (BMI), lower total cholesterol and triglyceride level. Blood pressure level was significantly lower in patients with reduced LnRHI. There was no significant difference regarding other parameters.

LnRHI was also compared between patients and age, sex-matched healthy controls. Although LnRHI tended to be lower in the patients than in the control subjects, the difference did not reach statistical significance (Patients: 0.52 ± 0.34; Controls: 0.59 ± 0.29; *p* = 0.14) (Table 2).

### Independent Clinical Determinants of LnRHI

In univariate analysis, LnRHI positively associated with phosphorus, SBP, DBP and negatively associated with triglyceride and calcium. These variables were further analyzed by multiple stepwise regression analysis and only DBP (ß = 0.006, *p* < 0.001) and calcium (ß = −0.183, *p* = 0.01) remained as independent determinants of LnRHI. The model explained 7% of the variance of LnRHI (Table 3).

### Associations between LnRHI and Conventional Endothelial Injury Markers

Table 4 presented the associations between LnRHI and conventional endothelial injury markers. In Model-1 with only age and sex adjusted, LnRHI showed no association with these markers except for tissue factor (ß = 0.12, *p* = 0.01). After adjusting for traditional cardiovascular risk factors in Model-2, the association between LnRHI and tissue factor became non-significant, with a marginal *p* value of 0.06. In the extensively adjusted Model-3 with dialysis vintage, interdialytic weight gain and log-CRP included as covariates, LnRHI was positively associated with vWF (ß = 0.09, *p* = 0.04) and tissue factor (ß = 0.09, *p* = 0.047).

### Associations between LnRHI and Cardiac Markers

Table 5 showed the associations between LnRHI and cardiac markers. There was a significant positive association between LnRHI and log-BNP in Model-1. However, after extensive adjustment (Model-2 and 3), the association disappeared. LnRHI showed no association with log-NT proBNP in any model. On the contrary, it was closely and positively associated with troponin levels in all three models.

## Discussion

Identifying novel cardiovascular risk markers is of critical importance for dialysis patients. Considering previous evidence from a variety of study populations^8^,^9^,^10^, it was natural to hypothesize that RHI is also a promising cardiovascular risk predictor in dialysis patients. To evaluate its value in this unique disease population, we performed this cross-sectional analysis with a focus on its performance and its cross-sectional correlators.

The existence of arteriovenous fistula access poses a special challenge for the performance of PAT test among dialysis patients. Obviously, the arm with the fistula should only be used as a control arm to avoid occlusion by the blood pressure cuff. Since a previous history of fistula-associated operation, even a failed one, could affect distal blood perfusion, we excluded those with previous operations for fistula creation on the non-access arm. However, even the fistula on the control arm could also have had unneglectable influences on the test result. First, fistula creation decreases digital perfusion pressure, thus may result in insufficient PAT signal^13^, as reflected by the high incidence of poor signal quality in our study. Second, the fistula may cause unsteady blood flow, e.g. turbulent flow, in the local circulation which can disrupt the steady PAT signal of the control arm and thus affect the calculation of RHI. Finally, the control arm serves as reflection of systemic state, but existence of the fistula may blunt the transduction of systemic fluctuation into distal circulation. Caution should be taken in interpreting our data since these probable influences cannot be avoided in the patients.

Unlike the general population, BP measurement before PAT test had to be done in the test arm. To avoid the affection of transient induction of NO release on the subsequent reactive hyperemia test, we stipulated that the test could only be performed no earlier than 20 minutes after BP measurement. Under these special restrictions, the test was successful in 88.9% subjects (311 out of 350). The main cause of a failed test was poor PAT signal, accounting for over 80% of the technically inadequate studies. This ratio was significantly higher than that reported in the Framingham cohort (30.9%) and the ELSA-Brasil cohort (54.0%)^14^,^15^. Therefore, signal quality is the major limitation of a successful PAT test in dialysis patients, even with strict eligibility restrictions. Among those with a successful test, the intraclass correlation coefficient analysis indicates that the reproducibility was moderate. Hence, the overall performance of the PAT test in dialysis patients is acceptable.

Unexpectedly, we found no statistically significant difference of LnRHI between dialysis patients and the control population, even though we noted that it tended to be lower in the dialysis patients. In a previous study by Hirata *et al*., the authors found a significantly lower LnRHI in CKD-patients (n = 383) compared with non-CKD patients (n = 474) (0.525 ± 0.194 vs. 0.588 ± 0.216, *p* < 0.001)^10^. It is worthy to note that the average LnRHI levels in their study were similar to ours, for both the patients and the controls. Therefore, a possible explanation for the absence of significant difference between patients and controls in our study is that we had limited number of the control subjects (n = 58).

Previous studies suggested that compared with FMD, RHI correlates more closely with metabolic factors^14^,^16^. In our analysis, this was partly confirmed as we found that dialysis patients with higher LnRHI tended to have lower BMI, lower total cholesterol and triglyceride levels. In addition, the linear regression analysis revealed that DBP (positively) and calcium (negatively) were independent determinants of LnRHI. However, it should be noted that the R^2^ of the model was only 0.07 and the regression coefficient for DBP is particularly low, suggesting that the associations were essentially clinically non-significant, and that this index is largely determined by unknown non-traditional clinical variables. In fact, studies on the determinants of RHI yielded widely varied and sometimes conflicting results and the abilities of their regression models for variance predicting were usually unsatisfied^14^,^15^,^17^,^18^,^19^,^20^. Hence more potent determinants of RHI, especially disease-specific factors, warrant further exploring.

The associations of RHI with several conventional endothelial injury markers were also evaluated in our study. We found that LnRHI had a modest association with vWF and tissue factor after extensive adjustment. These markers were widely used in different studies as surrogate measures of endothelial dysfunction. However, our results, together with previous studies, suggest that these markers and non-invasive endothelial function measures (both FMD and RHI) should not be regarded as interchangeable^21^,^22^.

Our analysis also revealed a paradoxically positive association between RHI and troponins. Elevated troponin levels are powerful prognostic predictors in dialysis patients^23^. Whether this indicates another “reverse epidemiology” phenomenon or implies that RHI and the markers respond to distinct pathophysiology process is unknown. In fact, confusing association between RHI and another cardiovascular risk marker, carotid artery intima-media thickness, has also been observed in the ELSA-Brasil cohort^24^. Future studies, especially those with hard outcomes, should pay attention to this question.

There are certain limitations in our study that should be noted. First, due to the cross-sectional design, we cannot establish any causality conclusions. Second, the exact effect of different dialysis access (type and location) on the PAT signal and RHI was not determined in our study, though we observed that poor signal was the major cause of a test failure. Third, although patients underwent repeated PAT testing on the same midweek nondialysis day during consecutive weeks, we did not obtain any measurement about their fluid status. Therefore the exact effect of volume status on RHI cannot be extrapolated from our study.

In conclusion, poor signal was the major cause of failed PAT test in dialysis patients. Traditional clinical variables had very limited influence on RHI in these subjects. Among a panel of conventional endothelial injury markers, RHI showed very modest associations with only vWF and tissue factor. RHI was positively associated with troponins in the patients. Our results showed that PAT testing is feasible in patients on hemodialysis. Future studies are warranted to explore the risk discrimination ability of RHI in these patients.

## Additional Information

**How to cite this article:** Wenjin, L. *et al*. Reactive Hyperemia Index in Patients on Maintenance Hemodialysis: Cross-sectional Data from a Cohort Study. *Sci. Rep.* **7**, 45757; doi: 10.1038/srep45757 (2017).

**Publisher's note:** Springer Nature remains neutral with regard to jurisdictional claims in published maps and institutional affiliations.

## Figures and Tables

**Table 1:** General Characteristics of the Patients dichotomized according to median LnRHI.

	LnRHI		*p*
	<0.50 (n = 150)	≥0.50 (n = 161)	
Age, years	53.3 ± 12.1	51.9 ± 12.3	0.31
Male	80 (53.3%)	95 (59.0%)	0.31
BMI, kg/m^2^	22.1 ± 3.5	21.3 ± 3.1	0.031*
Current Smoker	25 (16.7%)	37 (23.0%)	0.16
Dialysis Vintage, months	66.2 ± 45.4	62.4 ± 40.3	0.44
IDWG, kg	2.4 ± 0.9	2.6 ± 0.8	0.15
Diabetes	32 (21.3%)	23 (14.3%)	0.10
History of CVD	20 (13.3%)	13 (8.1%)	0.13
Use of Antihypertensives	109 (72.7%)	119 (73.9%)	0.80
Use of ACEI/ARB	38 (25.3%)	47 (29.2%)	0.45
Use of statins	6 (4.0%)	7 (4.3%)	0.88
Hemoglobin, g/L	108.2 ± 17.4	109.1 ± 16.6	0.62
Albumin, g/L	39.1 ± 4.5	39.5 ± 4.3	0.45
Total cholesterol, mmol/L	4.11 ± 0.95	3.87 ± 0.88	0.025*
Triglyceride, mmol/L	2.14 ± 1.43	1.77 ± 0.93	0.007*
HDL cholesterol, mmol/L	1.02 ± 0.28	0.99 ± 0.28	0.41
LDL cholesterol, mmol/L	2.11 ± 0.61	2.04 ± 0.60	0.31
Phosphorus, mmol/L	1.77 ± 0.50	1.87 ± 0.52	0.09
Calcium, mmol/L	2.30 ± 0.26	2.27 ± 0.26	0.30
Parathyroid hormone, pg/mL	290.3 (124.3–567.2)	273.0 (109.4–591.3)	0.78
CRP, ug/ml	5.40 (2.05–14.36)	5.35 (1.64–12.89)	0.68
SBP, mmHg	137.0 ± 21.2	143.6 ± 20.7	0.005*
DBP, mmHg	82.6 ± 11.7	88.2 ± 11.7	<0.001*
PP, mmHg	54.3 ± 16.2	55.4 ± 15.2	0.53
HR, bpm	75.9 ± 8.8	77.0 ± 10.2	0.33

^*^Indicates statistically significant.

Abbreviations: ACEI, angiotensin-converting enzyme inhibitor; ARB, angiotensin receptor blocker; BMI, body mass index; cfPWV, carotid-femoral pulse wave velocity; CRP, C-reactive protein; CVD, cardiovascular disease; DBP, diastolic blood pressure; HDL, high-density lipoprotein; HR, heart rate; IDWG, interdialytic weight gain; LDL, low-density lipoprotein; LnRHI, natural logarithm of reactive hyperemia index; PP, pulse pressure; SBP, systolic blood pressure.

**Table 2:** Clinical parameters and LnHRI in hemodialysis patients and healthy controls.

	Patients	Healthy Controls^$^	*p*
Age, years	52.6 ± 12.2	54.3 ± 9.0	0.31
Male	175 (56.3%)	33 (56.9%)	0.93
BMI, kg/m^2^	21.7 ± 3.3	25.6 ± 2.8	<0.001
Current Smoker	62 (19.9%)	11 (19.0%)	0.87
Diabetes	55 (17.7%)	2 (3.4%)	0.006
SBP	140.4 ± 21.2	124.9 ± 17.5	<0.001
DBP	85.5 ± 12.0	83.4 ± 11.2	0.20
LnRHI	0.52 ± 0.34	0.59 ± 0.29	0.14

^$^PAT test result was available for 58 subjects.

Abbreviation: BMI, body mass index; DBP, diastolic blood pressure; LnRHI, natural logarithm of reactive hyperemia index; SBP, systolic blood pressure.

**Table 3:** Independent Clinical Determinants of LnRHI in Dialysis Patients.

	ß	95% Confidence Interval	*p*
*Model R*^2^* = 0.07*			
Diastolic Blood Pressure	0.006	0.003–0.009	<0.001
Calcium	−0.183	−0.324 – −0.042	0.01

**Table 4:** Associations between LnRHI and Conventional Endothelial Injury Markers.

LnRHI	log-PAI1		log-vWF		log-Selectin		log-Tissue Factor		Thrombomodulin	
	ß	*p*	ß	*p*	ß	*p*	ß	*p*	ß	*p*
Model-1	−0.03	0.55	0.06	0.16	0.06	0.14	**0.12**	**0.01**	1.02	0.17
Model-2	0.04	0.36	0.08	0.07	0.05	0.29	0.08	0.06	0.74	0.33
Model-3	0.05	0.27	**0.09**	**0.04**	0.05	0.24	**0.09**	**0.047**	0.85	0.25

Model-1: adjusted for age and sex.

Model-2: adjusted for covariates in Model 1 + body mass index, smoking status, diabetes mellitus, history of cardiovascular disease, use of antihypertensives, use of statins, total and HDL cholesterol, triglyceride, mean arterial pressure.

Model-3: adjusted for covariates in Model 2 + dialysis vintage, interdialytic weight gain, log-CRP.

**Table 5:** Associations between LnRHI and Cardiac Markers.

LnRHI	log-BNP		log-NT proBNP		log-Troponin T		log-Troponin I	
	ß	*p*	ß	*p*	ß	*p*	ß	*p*
Model-1	**0.22**	**0.02**	0.01	0.93	**0.27**	**<0.001**	**0.26**	**0.005**
Model-2	0.14	0.13	−0.04	0.51	**0.24**	**<0.001**	**0.22**	**0.02**
Model-3	0.15	0.10	−0.03	0.62	**0.24**	**0.001**	**0.23**	**0.02**

Model-1: adjusted for age and sex.

Model-2: adjusted for covariates in Model 1 + body mass index, smoking status, diabetes mellitus, History of cardiovascular disease, use of antihypertensives, use of statins, total and HDL cholesterol, triglyceride, mean arterial pressure.

Model-3: adjusted for covariates in Model 2 + dialysis vintage, interdialytic weight gain, log-CRP.
